# Gepants for abortive treatment of migraine: A network meta‐analysis

**DOI:** 10.1002/brb3.1701

**Published:** 2020-06-11

**Authors:** Peiwei Hong, Tianlin Tan, Yao Liu, Jing Xiao

**Affiliations:** ^1^ Department of Geriatric Medicine and Neurology West China School of Public Health and West China Fourth Hospital Sichuan University Chengdu China; ^2^ Xindu Hospital of Traditional Chinese Medicine Chengdu China

**Keywords:** CGRP, gepants, migraine, network meta‐analysis

## Abstract

**Objectives:**

To evaluate and compare the efficacy and safety of gepants for abortive treatment of migraine by network meta‐analysis.

**Materials & Methods:**

Publications, which were randomized controlled trials (RCTs) about gepants for abortive treatment of migraine, were acquired from Pubmed and Cochrane Library. The literatures screening and quality assessment followed the Cochrane handbook. Review manager 5.3 and Addis v1.16.8 were utilized for data analyzing.

**Results:**

Totally, 15 RCTs were included in the network meta‐analysis. The trials enrolled were with high quality. There are 7 treatments were analyzed: BI 44370 TA, MK‐3207, olcegepant, rimegepant, telcagepant, ubrogepant, and placebo. Of these trials, 11,118 patients and 10,917 patients were assigned to one of 7 treatments randomly for efficacy assessment and safety assessment, respectively. In meta‐analysis of direct comparisons, all gepants were superior to placebo in achieving pain freedom 2 hr postdose and only rimegepant and telcagepant were higher than placebo in incidence of any adverse events. In network meta‐analysis, the rank best 3 drugs were olcegepant, BI 44370 TA, and MK‐3207 for efficacy outcomes. And the rank best 3 drugs were BI 44370 TA, placebo, and ubrogepant for safety outcomes.

**Conclusion:**

Gepants were effective for abortive treatment of migraine. The most effective treatment of gepants for migraine might be olcegepant which were administrated transvenously. And all of gepants were safe for migraine treatment with single dose.

## Graphical abstract

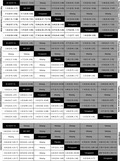

## 1 INTRODUCTION

Migraine disorder is the most common primary headache type which may influence nearly one‐seventh people worldwide (GBD 2016 Disease, & Injury Incidence & Prevalence Collaborators, 2016). It may affect the normal daily living and working of sufferers, even lead to paralysis (Headache Classification Committee of the International Headache Society (IHS), 2018). The treatments of migraine include abortive treatment and preventive treatment (Lambru, Andreou, Guglielmetti, & Martelletti, 2018). The most widely prescribed abortive treatment of migraine is triptans, which are the serotonin 5‐HT receptor agonists (Leroux & Rothrock, 2019). But triptans are not always effective for abortive treatment of migraine and with a high incidence of adverse events (Leroux and Rothrock, 2019). And the most serious adverse events are cardiovascular effects (Leroux & Rothrock, 2019).

Calcitonin gene‐related peptide (CGRP) is an important vasodilatory peptide which involved in migraine pathophysiology (Edvinsson, Haanes, Warfvinge, and Krause 2018; Messina & Goadsby, 2019; Edvinsson & Warfvinge, 2019). And its vasodilator effects could prevent myocardial ischemia, hypertension, and ischemic stroke (Edvinsson et al., 2018; Edvinsson & Warfvinge, 2019; Messina & Goadsby, 2019). Gepants, which are CGRP receptor antagonists, have been proven to be effective and safety for migraine of abortive treatment in some clinical trials (Connor et al., 2009; Croop et al., 2019; Diener et al., 2011; Dodick, Kost, Assaid, Lines, & Ho, 2011; Dodick et al., 2019; Hewitt, Aurora, et al., 2011; Hewitt, Martin, et al., 2011; Ho et al., 2010, 2012; Ho, Ferrari, et al., 2008; Ho, Mannix, et al., 2008; Lipton, Croop, et al., 2019; Lipton, Dodick, et al., 2019; Marcus et al., 2014; Olesen et al., 2004; Troconiz, Wolters, Tillmann, Schaefer, & Roth, 2006; Voss et al., 2016). In our previous study, we found that gepants were superior to placebo in efficacy outcomes according to meta‐analysis which did not distinguish the formulations (Han, Liu, & Xiong, 2019). And in the network meta‐analysis which conducted in late of 2018, the authors found that all of the gepants were superior to placebo in efficacy outcomes, and the more effective drug was olcegepant (Xu & Sun, 2019). Meanwhile, ubrogepant showed lower toxicity than other gepants. And there were 4 new randomized controlled trials(RCTs) had been published in 2019 which assessed the efficacy and safety of rimegepant and ubrogepant (Croop et al., 2019; Dodick et al., 2019; Lipton, Croop, et al., 2019; Lipton, Dodick, et al., 2019). But there are not gepants are approved for an acute treatment of migraine by Food and Drug Administration (FDA) so far. Here, we utilize the network meta‐analysis to analyze the efficacy and safety of gepants for an update, compared with placebo or one another gepants.

## 2 METHODS

### 2.1 Data selection

Database including Pubmed and Cochrane Library were queried using the following terms: migraine disorders, migraine without aura, migraine with aura, calcitonin gene‐related peptide, receptors, calcitonin gene‐related peptide, and calcitonin gene‐related peptide receptor antagonists. The searching results were filtered by a clinical trial. The cutoff date was December 15, 2019.

According to the PICO principle, the publications of RCTs published in English and matching the following criteria were enrolled: (a) the participants are diagnosed with migraine, (b) the interventions were gepants for an acute attack of migraine, (c) the comparisons were other gepants or placebo.

### 2.2 Data extraction and analysis

The procedure of data extraction and analysis was published in our previous publications (Hong & Liu, 2016). In brief, the assessing of risk of bias was followed with Cochrane collaboration' tool for evaluating risk of bias. The primary outcomes were incidence of pain freedom 2 hr postdose and any adverse events. The secondary outcomes were incidence of nausea freedom 2 hr postdose, phonophobia freedom 2 hr postdose, photophobia freedom 2 hr postdose, treatment‐related adverse events, abnormal liver function, and chest discomfort.

### 2.3 Statistical analysis

The direct comparisons between different gepants or placebo were analyzed by Review manager 5.3 (Cochrane Collaboration). *α* less than 0.05 was set as the significant level. The network meta‐analysis was conducted by Addis v1.16.8 (http://drugis.org/software/addis1/addis1.16) (Cipriani et al., 2009; Dias, Welton, Caldwell, & Ades, 2010; Xiao, Chen, Yang, & Kou, 2016). The software is designed according to the Bayesian hierarchical model and Markov Chain Monte Carlo (MCMC) method. The consistency of the network meta‐analysis was assessed by node‐splitting analysis (Dias et al., 2010). When *p* value was more than .05, the consistency model was chosen for drawing conclusions and ranking the included treatments. Otherwise, inconsistency model was utilized to analyze the data. Odds ratio (OR) and 95% confidence interval (CI) was selected as the effect magnitude.

### 2.4 Ethical statement

All of data analyzed in this article were from articles published, so the ethical approvement was not required.

## 3 RESULTS

**FIGURE 1:**
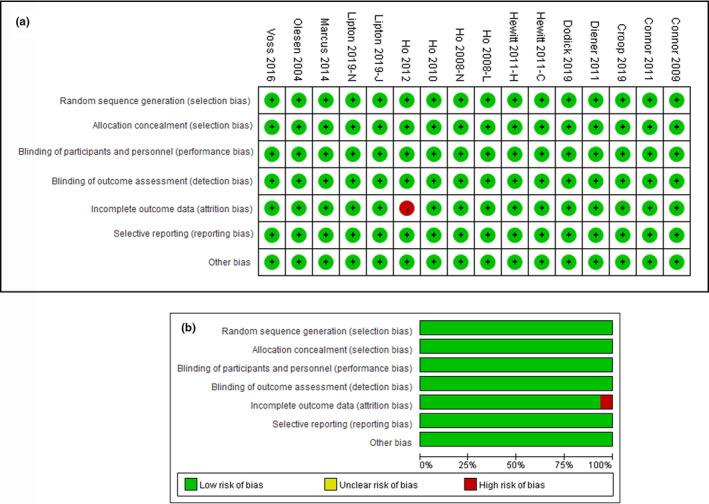
Risk of bias. (a) shows the review authors' judgments about each risk of bias item presented as percentages across all included studies. (b) shows review authors' judgments about each risk of bias item for each included study

Totally, we included 15 RCTs in the network meta‐analysis, after the removal of repetitions and unmatched publications. Six of 15 RCTs were phase 2 trials, the rest were phase 3 trials. Of these trials enrolled, 7 treatments were analyzed: BI 44370 TA, MK‐3207, olcegepant, rimegepant, telcagepant, ubrogepant, and placebo. All of the treatments were administrated with single dose. The doses of BI 44370 TA and olcegepant were 400 mg and 2.5 mg, respectively. The doses of MK‐3207 were range from 10 to 200 mg. The doses of rimegepant were range from 75 to 300 mg. The doses of telcagepant were range from 150 to 600 mg. The doses of ubrogepant were range from 25 to 100 mg. Most of gepants were administrated orally except olcegepant, which was administrated transvenously. The detail information was shown in Table 1. All of trials were two‐grouped studies. Of these trials, 11,118 patients and 10,917 patients were assigned to one of seven treatments randomly for efficacy assessment and safety assessment, respectively. The mean sample size was 1589 per group (range from 73 to 4,250) for efficacy assessment and 1,560 per group (range from 73 to 4,114) for safety assessment. Only one trial had high risk in incomplete outcome data (Ho et al., 2012). So, the quality of overall trails enrolled was good and their designs were similar. The risk of bias of trials enrolled was shown in Figure 1.

**TABLE 1:** The characteristic of randomized controlled trials enrolled

Study ID	Phase	Drug	Administration	Dosage	Outcomes
Olesen et al. (2004)	2a	Olcegepant	Intravenous infusion single dose	2.5 mg	①, ⑤, ⑦
Ho, Ferrari, et al. (2008)	3	Telcagepant	Oral single dose	150 mg/300 mg	①, ②, ③, ④, ⑤, ⑦, ⑧
Ho, Mannix, et al., (2008)	2	Telcagepant	Oral single dose	300, 400, and 600 mg	①, ②, ③, ④, ⑤, ⑥, ⑦
Connor et al. (2009)	3	Telcagepant	Oral single dose	150, 300 mg	①, ②, ③, ④, ⑤, ⑦
Ho et al. (2010)	3	Telcagepant	Oral single dose	140, 280 mg	①, ②, ③, ④, ⑤, ⑦
Diener et al. (2011)	2a	BI 44370 TA	Oral single dose	400 mg	①, ②, ③, ④, ⑤, ⑥, ⑦
Hewitt, Aurora, et al. (2011)	2a	MK‐3207	Oral single dose	10, 100, 200 mg	①, ②, ③, ④, ⑤, ⑥, ⑦
Hewitt, Martin, et al. (2011)	3	Telcagepant	Oral single dose	280 mg	①, ②, ③, ④, ⑤, ⑥, ⑦
Ho et al. (2012)	3	Telcagepant	Oral single dose	280 mg tablet/300 mg capsule	①, ②, ③, ④, ⑤, ⑥, ⑦, ⑧
Marcus et al. (2014)	2b	Rimegepant	Oral single dose	75, 150, and 300 mg	①, ②, ③, ④, ⑦, ⑧
Voss et al. (2016)	2b	Ubrogepant	Oral single dose	25, 50, and 100 mg	①, ②, ③, ④, ⑤, ⑥, ⑦, ⑧
Croop et al. (2019)	3	Rimegepant	Oral single dose	75 mg	①, ②, ③, ④, ⑤, ⑥, ⑦
Dodick et al. (2019)	3	Ubrogepant	Oral single dose	50 and 100 mg	①, ②, ③, ④, ⑤, ⑥, ⑦
Lipton, Croop, et al. (2019)	3	Rimegepant	Oral single dose	75 mg	①, ②, ③, ④, ⑤, ⑦
Lipton, Dodick, et al. (2019)	3	Ubrogepant	Oral single dose	25 and 50 mg	①, ②, ③, ④, ⑤, ⑥, ⑦

Note

①, Pain freedom 2 hr postdose; ②, Nausea freedom 2 hr postdose; ③, Phonophobia freedom 2 hr postdose; ④, Photophobia freedom 2 hr postdose; ⑤, Any adverse events; ⑥, Treatment‐related adverse events; ⑦, Abnormal liver function; ⑧, Chest discomfort.

**FIGURE 2:**
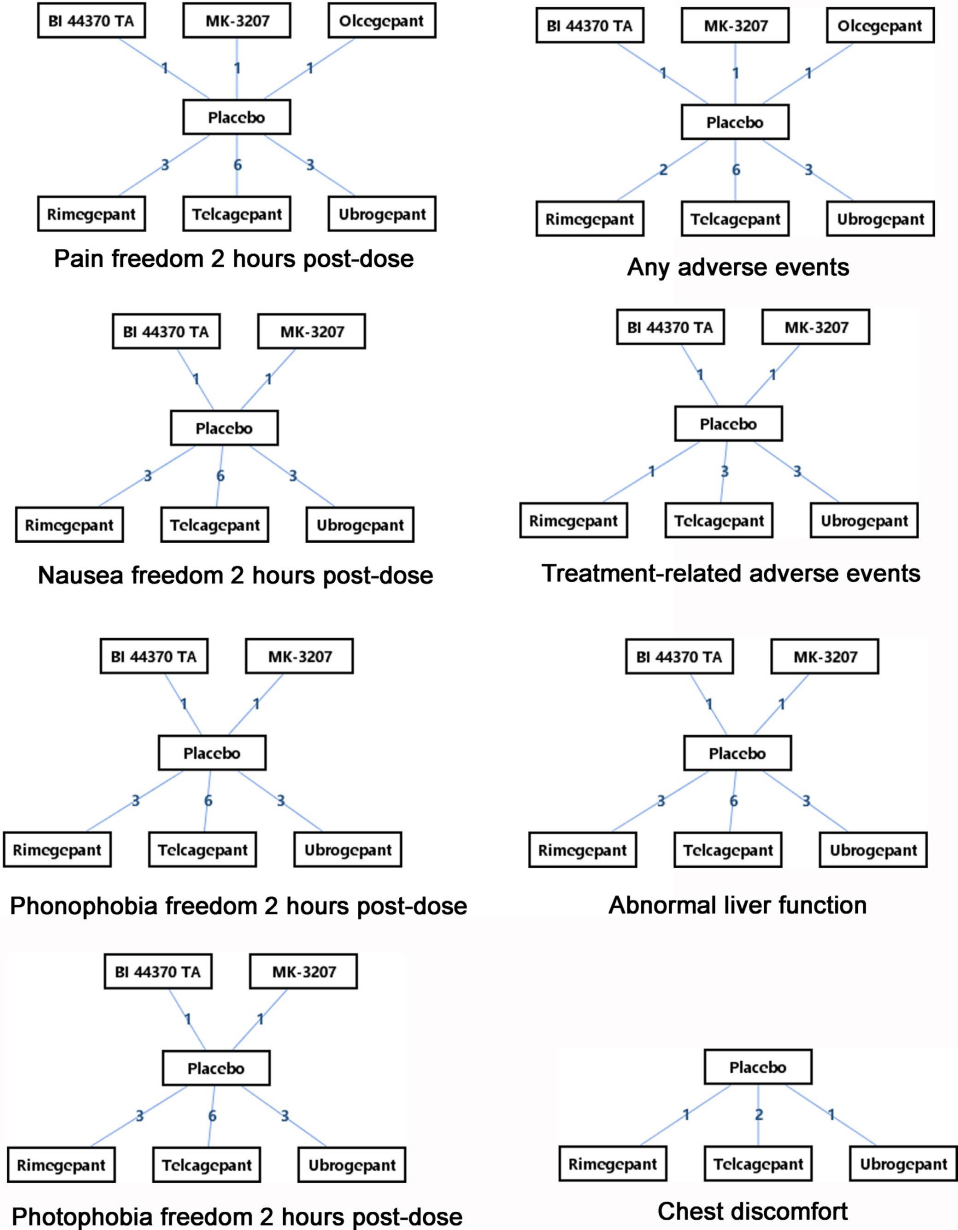
Network of eligible CGRP antagonists for the network meta‐analysis. The Arabic numerals between two drugs mean number of randomized controlled trials enrolled to compare the two drugs

Figure 2 showed the network of comparisons for efficacy/safety.

All gepants had one placebo‐controlled randomized trial at least, but there no existed head to head comparisons between gepants.

### 3.1 Efficacy

Regarding primary efficacy outcome (pain freedom 2 hr postdose), the heterogeneity was no exist between 6 pair‐wise comparisons. And all gepants were superior to placebo in meta‐analysis of direct comparisons (Table 2). In the network meta‐analysis, olcegepant was the rank 1 gepants to achieve pain freedom. And the next two were BI 44370 TA and MK‐3207(Table 3).

**TABLE 2:** Summary estimates for efficacy and acceptability in meta‐analysis of direct comparisons between CGRP antagonists or placebo

Comparisons	BI 44370 TA versus Placebo	MK‐3207 versus Placebo	Olcegepant versus Placebo	Rimegepant versus Placebo	Telcagepant versus Placebo	Ubrogepant versus Placebo
Pain freedom 2 hr postdose	**4.03 [1.51, 10.75]**	**3.65 [1.89, 7.04]**	**31.11 [3.80, 254.98]**	**2.11 [1.72, 2.58]**	**2.64 [2.20, 3.17]**	**1.85 [1.49, 2.28]**
Nausea freedom 2 hr postdose	**2.75 [1.39, 5.47]**	1.44 [0.90, 2.29]	Missing	**1.36 [1.14, 1.62]**	**1.67 [1.47, 1.90]**	**1.24 [1.06, 1.46]**
Phonophobia freedom 2 hr postdose	**2.41 [1.23, 4.72]**	**1.78 [1.13, 2.81]**	Missing	**1.84 [1.41, 2.39]** ^a^	**1.75 [1.54, 1.98]**	**1.44 [1.24, 1.68]**
Photophobia freedom 2 hr postdose	**2.62 [1.33, 5.17]**	**1.64 [1.04, 2.59]**	Missing	**1.84 [1.56, 2.19]**	**1.83 [1.49, 2.24]** ^a^	**1.57 [1.23, 2.00]** ^a^
Any adverse events	0.95 [0.32, 2.88]	1.50 [0.89, 2.51]	2.40 [0.70, 8.22]	**1.27 [1.01, 1.60]**	**1.17 [1.02, 1.33]**	1.03 [0.83, 1.28]
Treatment‐related adverse events	0.96 [0.06, 15.62]	1.44 [0.72, 2.87]	Missing	1.35 [0.86, 2.11]	1.35 [0.83, 2.18]	1.08 [0.84, 1.40]
Abnormal liver function	Not estimable	Not estimable	Not estimable	1.05 [0.50, 2.19]	1.08 [0.07, 17.45]	2.05 [0.52, 8.14]
Chest discomfort	Missing	Missing	Missing	Not estimable	2.43 [0.41, 14.37]	2.57 [0.13, 50.09]

Note

Values in bold means significant difference.

^a^ *I* ^2^ > 50%, and random‐effect model was utilized to estimate effect magnitude.

**TABLE 3:** Rank probability of efficacy of gepants

Drug	Rank 1	Rank 2	Rank 3	Rank 4	Rank 5	Rank 6	Rank 7
Pain freedom 2 hr postdose							
BI 44370 TA	0.02	0.53	0.25	0.08	0.04	0.07	0.01
MK‐3207	0.01	0.41	0.43	0.1	0.03	0.02	0
Olcegepant	0.97	0.02	0	0	0	0	0
Placebo	0	0	0	0	0	0.01	0.99
Rimegepant	0	0	0.02	0.15	0.6	0.22	0
Telcagepant	0	0.04	0.28	0.6	0.07	0.01	0
Ubrogepant	0	0	0.01	0.07	0.25	0.67	0

Drug	Rank 1	Rank 2	Rank 3	Rank 4	Rank 5	Rank 6
Nausea freedom 2 hr postdose						
BI 44370 TA	0.5	0.18	0.1	0.06	0.06	0.1
MK‐3207	0.23	0.2	0.13	0.09	0.1	0.25
Placebo	0.01	0.05	0.18	0.34	0.32	0.11
Rimegepant	0.13	0.25	0.22	0.15	0.13	0.12
Telcagepant	0.03	0.11	0.17	0.2	0.24	0.25
Ubrogepant	0.11	0.21	0.21	0.16	0.14	0.16
Phonophobia freedom 2 hr postdose						
BI 44370 TA	0.66	0.12	0.06	0.09	0.06	0.01
MK‐3207	0.17	0.26	0.15	0.19	0.21	0.02
Placebo	0	0	0	0	0.03	0.97
Rimegepant	0.11	0.34	0.29	0.21	0.05	0
Telcagepant	0.06	0.26	0.42	0.23	0.04	0
Ubrogepant	0.01	0.02	0.08	0.28	0.61	0
Photophobia freedom 2 hr postdose						
BI 44370 TA	0.69	0.12	0.09	0.06	0.04	0.01
MK‐3207	0.11	0.16	0.14	0.19	0.36	0.04
Placebo	0	0	0	0	0.05	0.95
Rimegepant	0.15	0.47	0.26	0.09	0.03	0
Telcagepant	0.04	0.21	0.39	0.29	0.07	0
Ubrogepant	0.01	0.05	0.13	0.36	0.45	0

Note

Rank 1 is best and rank *N* is worst.

Regarding the secondary outcomes, the comparison between olcegepant and placebo was missing. In nausea freedom 2 hr postdose, all gepants were superior to placebo except MK‐3207. And the rank best drug was BI 44370 TA, the next two were rimegepant and ubrogepant. All of gepants were superior to placebo in achieving phonophobia freedom 2 hr postdose and photophobia freedom 2 hr postdose. And in the network meta‐analysis of phonophobia freedom 2 hr postdose, the rank best 3 were BI 44370 TA, rimegepant, and telcagepant. Meanwhile, in the photophobia freedom 2 hr postdose, the rank best 3 were BI 44370 TA, rimegepant, and telcagepant also. The detail information was showed in Tables 2 and 3.

### 3.2 Safety

Regarding primary safety outcomes, only rimegepant and telcagepant were higher than placebo in incidence of any adverse events in pair‐wise meta‐analysis (Table 2). And in the network meta‐analysis, the rank best 3 drugs were BI 44370 TA, placebo, and ubrogepant (Table 4).

**TABLE 4:** Rank probability of acceptability of gepants

Drug	Rank 1	Rank 2	Rank 3	Rank 4	Rank 5	Rank 6	Rank 7
Any adverse events							
BI 44370 TA	0.05	0.16	0.1	0.05	0.06	0.06	0.52
MK‐3207	0.2	0.45	0.16	0.08	0.04	0.04	0.03
Olcegepant	0.7	0.13	0.05	0.03	0.02	0.03	0.04
Placebo	0	0	0.01	0.05	0.24	0.49	0.22
Rimegepant	0.03	0.18	0.35	0.26	0.12	0.04	0.02
Telcagepant	0.01	0.06	0.26	0.36	0.23	0.06	0.01
Ubrogepant	0	0.02	0.07	0.18	0.3	0.27	0.17

Drug	Rank 1	Rank 2	Rank 3	Rank 4	Rank 5	Rank 6
Treatment‐related adverse events						
BI 44370 TA	0.35	0.09	0.05	0.04	0.05	0.41
MK‐3207	0.22	0.23	0.17	0.11	0.16	0.11
Placebo	0	0.02	0.11	0.3	0.35	0.21
Rimegepant	0.17	0.22	0.23	0.15	0.13	0.1
Telcagepant	0.23	0.34	0.2	0.14	0.06	0.03
Ubrogepant	0.03	0.1	0.23	0.26	0.25	0.14
Abnormal liver function						
BI 44370 TA	0.24	0.06	0.02	0.03	0.24	0.42
MK‐3207	0.22	0.06	0.02	0.02	0.22	0.47
Placebo	0.01	0.17	0.37	0.32	0.12	0.02
Rimegepant	0.06	0.19	0.31	0.28	0.13	0.03
Telcagepant	0.1	0.15	0.13	0.29	0.27	0.07
Ubrogepant	0.38	0.37	0.16	0.06	0.02	0

Drug	Rank 1	Rank 2	Rank 3	Rank 4
Chest discomfort				
Placebo	0	0.08	0.69	0.24
Rimegepant	0.11	0.11	0.04	0.73
Telcagepant	0.09	0.66	0.24	0.02
Ubrogepant	0.8	0.15	0.04	0.01

Note

Rank 1 is worst and rank *N* is best.

Regarding secondary safety outcomes, the comparison of olcegepant and placebo was missing in treatment‐related adverse events. And there were no differences between all gepants and placebo. And in the network meta‐analysis, the rank best 3 drugs were BI 44370 TA, placebo, and ubrogepant. In the incidence of abnormal liver function, the comparisons between BI 44370 TA and placebo, MK‐3207 and placebo or olcegepant and placebo were not estimable, because the number of patients suffered from abnormal liver function was zero. And there were no differences between the rest gepants and placebo. In the incidence of chest discomfort, the trials about BI 44370 TA, MK‐3207, and olcegepant had not reported this event. And the comparisons between rimegepant and placebo were not estimable, because the number of patients suffered from chest discomfort was zero. So, the network meta‐analysis of abnormal liver function and chest discomfort was hard to draw a conclusion. The detail information was showed in Tables 2 and 4.

**FIGURE 3:**
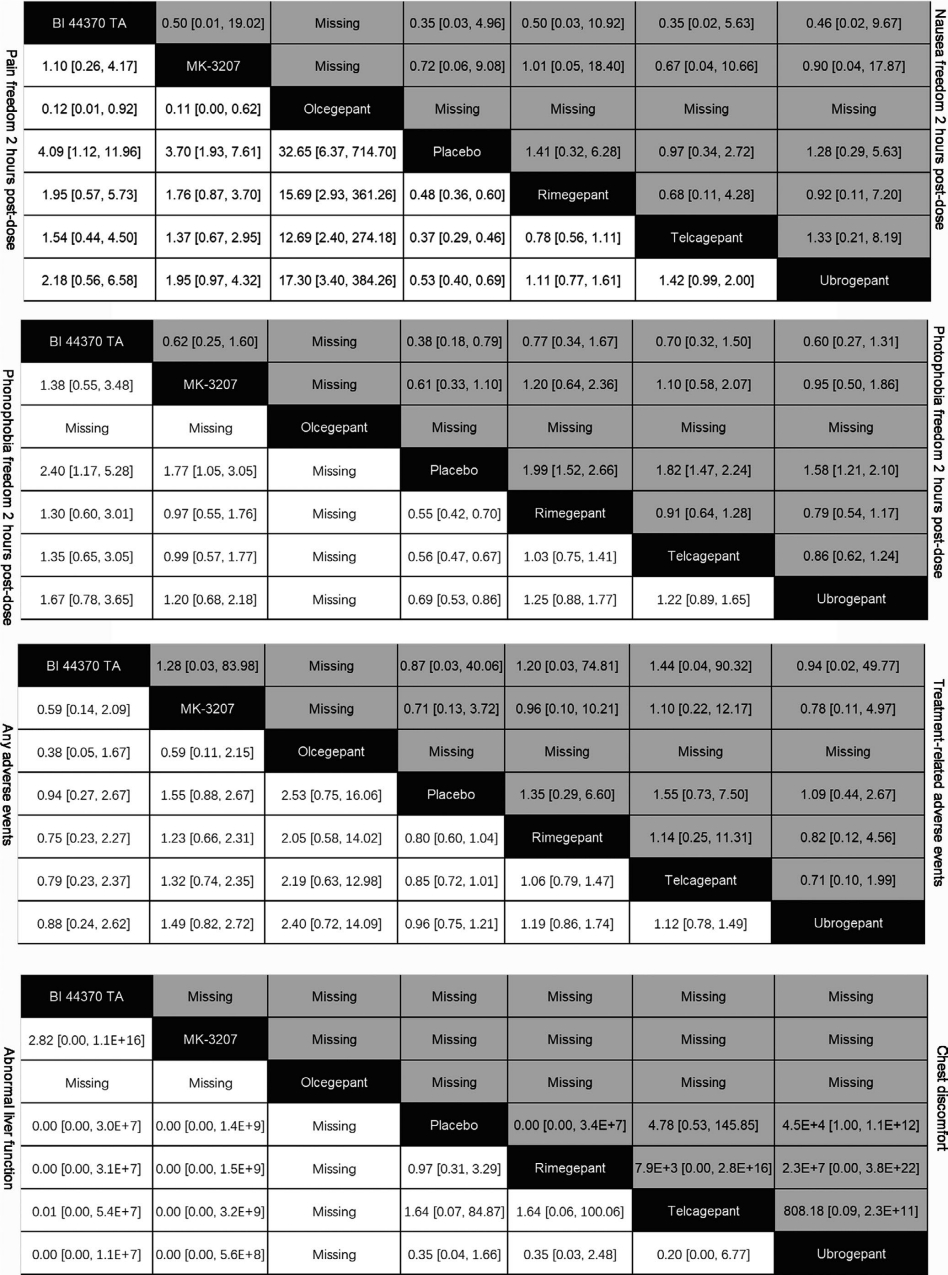
Network meta‐analysis of efficacy and acceptability of CGRP antagonists for migraine. The odds ratios (ORs) of comparisons of drugs are between the column‐defining drug and the row‐defining drug. Regarding efficacy, ORs higher than 1 favors the column‐defining drug. Regarding acceptability, ORs lower than 1 favors the column‐defining drug

Figure 3 showed the estimate effect values of different comparisons.

## 4 DISCUSSION

Our study showed that all of gepants with different dosages and administrated once were effective and safe for abortive treatment for migraine based on the results of 15 RCTs with high quality. And our results might help clinicians to choose the type and dosage of gepants. In terms of primary efficacy, all gepants were superior to placebo, and the most effective of gepants was olcegepant according to network meta‐analysis. Regarding to primary safety outcome, only rimegepant and telcagepant were inferior to placebo, but there are no existed fatal adverse events in gepants group.

In the meta‐analysis, we assessed the efficacy of gepants for improving associated symptoms of migraine. It was a pity that the data of olcegepant about associated symptoms of migraine was missing. And we found that most gegpants could improve nausea except MK‐3207, which nausea was its drug‐related adverse event (Hewitt, Aurora, et al., 2011). And the best gepants to achieve nausea freedom was BI 44370 TA. All of gepants could improve phonophobia and photophobia, and the best gepants to achieve phonophobia freedom and photophobia freedom 2 hr postdose were BI 44370 TA. So, the best gepants to improve associated symptoms of migraine was BI 44370 TA.

Concerning the safety of gepants, although the incidence of any adverse events of rimegepant and telcagepant was higher than placebo, but there was no difference between gepants and placebo in treatment‐related adverse events. There are concerns about potential cardiovascular risk after CGRP blockade. Olcegepant, a gepants administrated intravenously, had not reported the incidence of cardiovascular events (Olesen et al., 2004). And so on BI 44370 TA and MK‐3207 (Diener et al., 2011; Hewitt, Aurora, et al., 2011). These three gepants were discontinued because of different reasons. Telcagepant, which were evaluated in some clinical trials about abortive treatment of migraine, had not reported cardiovascular events (Connor et al., 2011; Connor et al., 2009; Hewitt, Martin, et al., 2011; Ho et al., 2010, 2012; Ho, Ferrari, et al., 2008; Ho, Mannix, et al., 2008). But it was discontinued because of liver enzymes level increment after repeat use (Negro & Martelletti, 2019). Rimegepant, which was called BMS‐927711, were evaluated in migraineurs in some clinical trials (Croop et al., 2019; Lipton, Croop, et al., 2019; Marcus et al., 2014). In a phase 2b trials, rimegepant were administrated orally with different dosages (range from 10 to 600 mg) (Marcus et al., 2014). And there were no cardiovascular events which were verified by ECG in rimegepant (Marcus et al., 2014). In two phase 3 clinical trials had not reported the cardiovascular events when rimegepant were administrated with 75 mg orally (Croop et al., 2019; Lipton, Croop, et al., 2019). But one patient in rimegepant group experienced transaminase concentration greater than 3 fold of the upper limit of normal (ULN) (Croop et al., 2019). And 13 patients suffered from transient transaminase concentration increasing in rimegepant group, but there were no difference between placebo and rimegepant group (Lipton, Croop, et al., 2019). Ubrogepant, which was distinct from MK‐3207 and telcagepant, were evaluated in migraine for acute treatment. And there are three patients suffered from chest discomfort and 1 patient experienced chest pain after ubrogepant treatment (Dodick et al., 2019; Lipton, Dodick, et al., 2019; Voss et al., 2016). And there were 11 patients experienced liver function lesion which transaminase concentration greater than 3 fold of ULN (Dodick et al., 2019; Lipton, Dodick, et al., 2019; Voss et al., 2016). And in our meta‐analysis, the incidence of abnormal liver function and chest discomfort were no differences between gepants and placebo, which were consistence with original trials. So, gepants with single dose were safety for an abortive treatment of migraine.

The limitations of present study were as follow. Firstly, the follow‐up period of trials enrolled was short, and the results of safety might be underestimated. Secondly, the sample sizes of different gepants were varied widely, which might affect the rank of gepants. Finally, our results apply only to abortive treatment of migraine and have not offered the preventive treatment of migraine.

In conclusion, gepants were effective for abortive treatment of migraine. The most effective treatment of gepants for migraine might be olcegepant which were administrated transvenously. And all of gepants were safe for migraine treatment with single dose.

## CONFLICT OF INTEREST

None.

## AUTHORS CONTRIBUTIONS

Peiwei Hong and Jing Xiao put forward the idea. Peiwei Hong, Tianlin Tan, and Yao Liu acquired the data. Peiwei Hong and Jing Xiao analyzed the data and wrote the first draft. Tianlin Tan and Yao Liu revised the draft.

## Data Availability

All of data were extracted from previously published data.
